# A Rigorous Temperature-Dependent Stochastic Modelling and Testing for MEMS-Based Inertial Sensor Errors

**DOI:** 10.3390/s91108473

**Published:** 2009-10-27

**Authors:** Mohammed El-Diasty, Spiros Pagiatakis

**Affiliations:** Department of Earth and Space Science and Engineering, York University, Toronto, ON M3J 1P3, Canada; E-Mail: spiros@yorku.ca

**Keywords:** MEMS, inertial sensor, temperature, AR model, GM model, UKF

## Abstract

In this paper, we examine the effect of changing the temperature points on MEMS-based inertial sensor random error. We collect static data under different temperature points using a MEMS-based inertial sensor mounted inside a thermal chamber. Rigorous stochastic models, namely Autoregressive-based Gauss-Markov (AR-based GM) models are developed to describe the random error behaviour. The proposed AR-based GM model is initially applied to short stationary inertial data to develop the stochastic model parameters (correlation times). It is shown that the stochastic model parameters of a MEMS-based inertial unit, namely the ADIS16364, are temperature dependent. In addition, field kinematic test data collected at about 17 °C are used to test the performance of the stochastic models at different temperature points in the filtering stage using Unscented Kalman Filter (UKF). It is shown that the stochastic model developed at 20 °C provides a more accurate inertial navigation solution than the ones obtained from the stochastic models developed at −40 °C, −20 °C, 0 °C, +40 °C, and +60 °C. The temperature dependence of the stochastic model is significant and should be considered at all times to obtain optimal navigation solution for MEMS-based INS/GPS integration.

## 1. Introduction

The performance of an integrated Global Positioning System (GPS)/Inertial Navigation System (INS) is mainly characterised by the ability of the INS to bridge GPS outages. In recent years, a promising technology namely, Micro-Electro-Mechanical Systems (MEMS)-based inertial sensors, has been developed, which can provide a low-cost navigation solution when integrated with GPS. MEMS systems are commonly fabricated using silicon, which possesses significant electrical and mechanical advantages over other materials [1]. However, due to the small size and weight of the MEMS-based inertial units, their performance characteristics are highly dependent on the temperature variations. Since these errors accumulate over time, the navigation solution degrades if the temperature effects on both, accelerometer and gyroscope (biases and scale factors) are not modelled and compensated [2]. Hence, there is a need for the development of accurate, reliable rigorous thermal models to reduce the effect of these temperature variations on the inertial sensor errors.

The inertial sensor errors can be divided into two types: deterministic (systematic) errors and random errors [3]. If not treated, such errors cause a rapid degradation in the INS navigation solution during the GPS outage period. In order to integrate MEMS inertial sensors with GPS, and to provide a continuous and reliable integrated navigation solution, the characteristics of different error sources and the understanding of the stochastic characteristics of these errors are of significant importance [4].

The deterministic error sources include bias and scale factor errors, which can be removed by specific calibration procedures in a laboratory environment. Park and Gao [4] discussed the laboratory calibration procedure for MEMS units, whereas Shin and El-Sheimy [5] developed field calibration procedures. Abdel-Hamid [6] implemented the deterministic error (bias and scale factor) to MEMS IMU at different temperature points and demonstrated that the deterministic error is temperature-dependent. Aggarwal *et al.*, [7] investigated the use of a simple polynomial temperature model to compensate for the inertial bias and scale factor deterministic errors and concluded that the inertial navigation solution was significantly improved.

On the other hand, the inertial sensor random errors primarily include the sensor noise, which consists of two parts, a high and a low frequency component. The high frequency component has white noise characteristics, while the low-frequency component is characterised by correlated noise [8]. A de-noising methodology is required to filter out the high frequency noise of the inertial sensor measurements prior to processing, using a low pass filter or a wavelet de-noising technique [3-6-8-9]. However, the low frequency noise component (correlated noise) can be modelled with sufficient accuracy using random processes [3] such as, random constant (random bias), random walk, Gauss-Markov or periodic random processes. Details of these stochastic models can be found in Nassar [3] and Gelb [10]. The most commonly used process is the first order Gauss-Markov process, whereas more recently, the use of Auto-Regressive (AR) modelling methods on low cost sensors were tested (e.g., Nassar, [3]; Park and Gao, [4]). Moreover, Hou and El-Sheimy [11] used Allan variance to study the random error of MEMS-based IMU, and demonstrated that the most dominant error has random walk characteristics.

A specific shortcoming in most of the above investigations is the disregard of the stochastic variation of these errors, which is of significant importance, and has not yet been investigated at different temperature points. The GPS/INS integrated system accuracy is significantly affected by the stochastic characteristics of the inertial navigation system [12]. Traditionally, the inertial navigation error model consists of three position errors, three velocity errors, and three attitude errors in addition to the, three gyro and three accelerometer bias errors. The process of understanding the stochastic variation of the errors at different temperature points is one of the most important steps for developing a reliable low-cost integrated navigation system. The reason is that a low-cost IMU accumulates relatively large navigation errors in a small time interval. Unless an accurate temperature-dependent stochastic model is developed, the mechanisation parameters will possess larger errors that could significantly degrade the system performance. Therefore, there is a need for the development of accurate, reliable and rigorous stochastic models, which can be used in the INS/GPS filter to provide an accurate navigation solution [3-12].

This paper examines the effect of changing the temperature points on the MEMS inertial sensor noise models using for the first time a rigorous Autoregressive-based Gauss-Markov process (AR-based GM). In this work we collect static data sets under different temperature points using a MEMS-based IMU, namely the ADIS16364 [13] and we use them to develop AR-based GM stochastic models at different temperature points. In addition, field kinematic test data collected at about 17 °C are used to test the performance of the stochastic models at different temperature points in the filtering stage when using Unscented Kalman Filter (UKF) with GPS position and heading updates. It should be noted that the focus of this paper is to investigate the effect of the IMU temperature variations on the navigation solution and therefore either UKF or Extended-KF (EKF) can be used. It has been demonstrated in the scientific literature (see Wendel *et. al.* [14] for example) that the UKF and EKF show very similar performance and thus, testing UKF and EKF algorithms is not of concern in this paper.

## 2. Rigorous Autoregressive-Based Gauss-Markov Model

In this section we briefly describe the Gauss-Markov (GM), and Autoregressive (AR) models and then we derive the AR-based GM model. For more details on stochastic modelling of inertial sensor errors see El-Diasty and Pagiatakis [15].

### 2.1. Gauss-Markov Model

Gauss-Markov (GM) random processes are stationary processes that have exponential autocorrelation functions. GM processes are important because they represent a large number of physical processes with reasonable accuracy, while they exhibit a relatively simple mathematical formulation [10]. A stationary Gaussian process that has an exponentially decaying autocorrelation is called first-order GM process. For a random process *x* with zero mean, mean square error $\sigma_{\text{w}}^{2}$, and correlation time T_c_, the model is described by the following continuous equation of time [10-15]:

(1) $$\dot{x}=-\frac{\text{1}}{T_{c}}x+w$$

**Figure 1.**
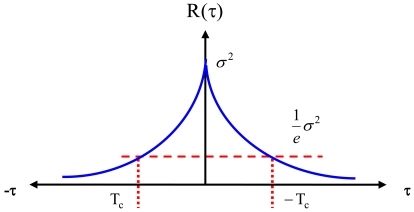
Autocorrelation function of the first-order Gauss-Markov process.

The autocorrelation function (see Figure 1) of the GM model is given by [10-15]:

(2) $$R\left(\tau \right)=E\left[x\left(t\right)x\left(t+\tau \right)\right]=\sigma^{2}e^{-\left|\tau \right|/T_{C}}$$

where *τ* is the time shift, *T_c_* is the correlation time, and *σ*^2^ is the variance at zero time shift (τ = 0). The most important characteristic of the GM process is that it can represent bounded uncertainty, which means that any correlation coefficient at any time shift is less or equal to the correlation coefficient at zero time shift *R*(*τ*) ≤ *R*(0) for all *τ* [10-15]. Two parameters namely, T_c_ (correlation time) and $\sigma_{w}^{2}$ (driven noise variance), are required to describe a first-order GM process, as shown in Figure 1.

The discrete time model of GM process can be written as [15-16]:

(3) $$x_{k}=e^{-\Delta t/T_{c}}x_{k-1}+w_{k}$$

and the associated variance can be estimated by using the following formula [15-16]:

(4) $$\sigma_{w_{k}}^{2}=\sigma_{x_{k}}^{2}\left(1-e^{{-2\Delta t_{k}/T}_{C}}\right)$$

Thus, the discrete-time first-order GM model can take the form of Equation (3) and the variance of the driven noise *w_k_* is given by Equation (4). The first-order GM process has been widely used in inertial navigation filters because of its bounded uncertainty characteristic that makes it the best model for slowly varying sensor errors, such as residual bias and scale errors [15-17]. The first-order Gauss-Markov model parameters can be estimated using least squares fitting of the estimated autocorrelation values for gyro and accelerometer measurements. However, inaccurate GM modelling of the inertial sensor random errors is most likely expected due to inaccurate autocorrelation function determination [3-12].

### 2.2. Autoregressive Model

**Figure 2.**
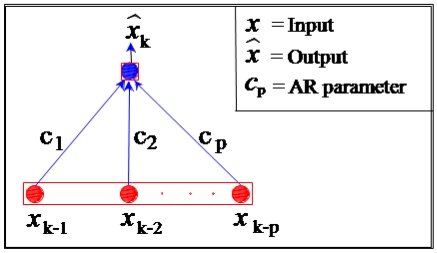
Autoregressive (AR) structure.

To avoid the problem of inaccurate modelling of inertial sensor random errors due to inaccurate autocorrelation function determination, we can apply another method for estimating inertial sensor errors, as introduced by Nassar [3]. Compared to a first-order GM random process, Autoregressive (AR) processes have more modelling flexibility since they are not always restricted to only one parameter, and higher orders can be used [3]. In many time series applications, AR processes are used to model (estimate) their stochastic part [10]. The inertial sensor data are considered to form a time series that contain both, systematic and stochastic error components, and hence, AR models are used to describe the inertial stochastic errors. The GM process given by Equation (3) is equivalent to an AR process of first-order [3-12]. An AR process is a time series produced by a linear combination of past values and its structure is shown in Figure 2 [15].

An AR process of order *p* can be described by the following linear equation [15-18]:

(5) $${\hat{x}}_{k}=\sum_{i=1}^{p}c_{i}x_{k-i}+w_{k},$$

where *x̂_k_* is the process output, *x_k-i_* are previous system states, and *c_i_* are the AR model parameters. The AR model parameters can be estimated using least-squares fitting [12] or can alternatively be estimated using Yule-Walker, covariance and Burg's methods [3]. The variance of the noise component *w_k_* (is also equivalent to the mean square error MSE in this case because the expected mean of the residual is equal zero) can be estimated numerically from the following equation [15-18]:

(6) $$\sigma_{w_{k}}^{2}=\frac{1}{n}{\sum_{k=1}^{n}\left(x_{k}^{d}-{\hat{x}}_{k}\right)}^{2}$$

where *n* is the size of the sample of the stationary dataset, $x_{k}^{d}$ is the known value of the process (desired output), and *x̂_k_* is the corresponding estimated output.

If we have a first-order AR model, then the discrete form will be [15-18]:

(7) $$x_{k}=c_{1}\cdot x_{k-1}+w_{k},$$

for which the associated variance of the noise component *w_k_* can numerically be estimated from stationary data using Equation (6). The AR model was introduced by Nassar [3] as an alternative to GM process for the modelling of the gyro residuals and accelerometer biases. Also, El-Diasty *et al.* [19] showed that the first-order AR model is a statistically significant process for modelling MEMS-based inertial sensor errors. However, the only disadvantage of AR model is that it does not include the sampling interval, which is not constant in inertial navigation systems due to the inadequacy of the data acquisition system to capture the high sampling rates of the IMU sensor output.

### 2.3. Rigorous Autoregressive-Based Gauss-Markov Model

To take the advantage of both, the AR and GM models, we choose the first-order GM model [Equation (3)] in which the sampling rate is considered, whereas we estimate the correlation time *T_c_* from the AR parameter *c*_1_ and not from the autocorrelation function approximation. This is possible by combining Equation (3) and Equation (7), (equating their right-hand sides). This gives:

(8) $$e^{-\Delta t_{k+1}/T_{c}}=c_{1}$$

If we take the natural logarithm of both sides, then:

(9) $$\frac{-\Delta t_{k+1}}{T_{c}}=\ln(c_{1}).$$

Therefore, the correlation time can be estimated from first-order AR model parameter *c*_1_ as follows:

(10) $$T_{c}=\frac{-\Delta t_{k+1}}{\ln(c_{1}).}$$

**Figure 3.**
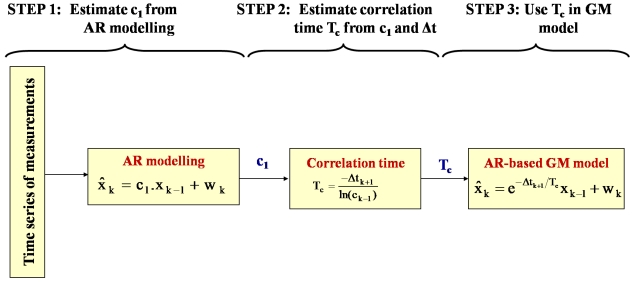
AR-based GM stochastic modelling steps.

In this paper, we call this process AR-based GM model. Figure 3 shows the steps for building the AR-based GM model. The AR-based GM model is proposed in this paper for two reasons: a) in an AR-based GM model, a short stationery data set can be used to estimate the model correlation time, whereas the traditional GM-only model needs a very long data set, which should equal 200 times the expected correlation time with 10% uncertainty according to Nassar [3], and b) in an AR-based GM model, the sampling interval can be accounted for whereas in an AR-only model the sampling interval is not considered at all and unequally spaced data that are so common in real IMUs experiments can definitely introduce errors in the solution and hence can be considered as sub-optimal navigation solution. In this paper, we reckon that the AR-based GM model is the only correct model to use when unequally spaced data are available in addition to being simple and feasible when using short data sets.

## 3. Test Description

**Figure 4.**
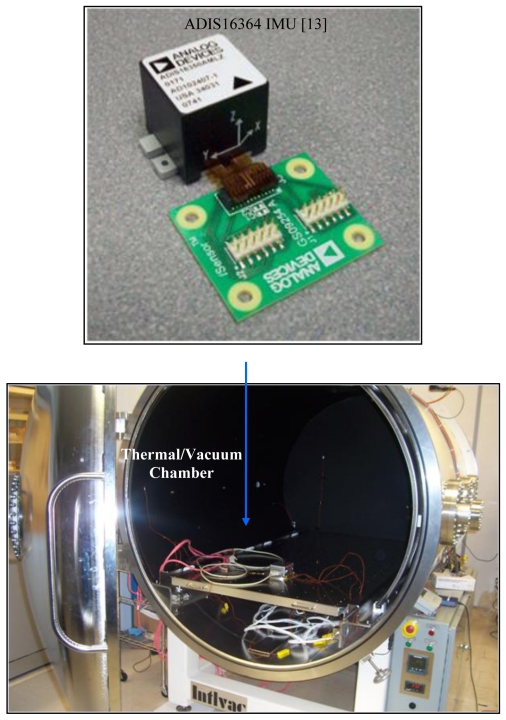
The thermal/vacuum chamber and the position of the IMU during testing.

Figure 4 shows pictures of the static test setup. The data were collected at the Space Instrumentation Laboratory (SIL) of York University, which, among others, is equipped with a thermal/vacuum chamber. Static data sets were collected under different temperature points using the ADIS16364 inertial measurement unit (IMU) from Analog Devices Inc. [13] (see Table 1 for the specifications of the ADIS16364 IMU). The ADIS16364 IMU static data were collected with a sampling rate of 200 Hz at different temperature points in the range −40 °C to +60 °C with 20 °C step. Thus, the performed test covers the operational temperature of the ADIS16364 IMU.

**Figure 5.**
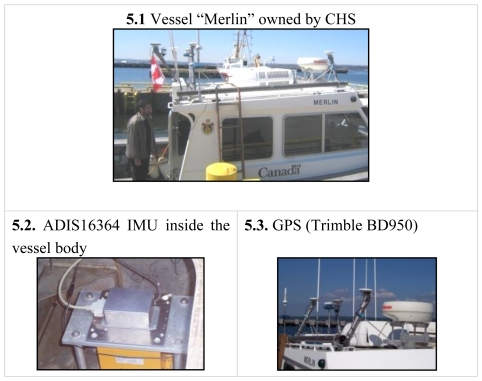
Field system used to collect kinematic data.

**Figure 6.**
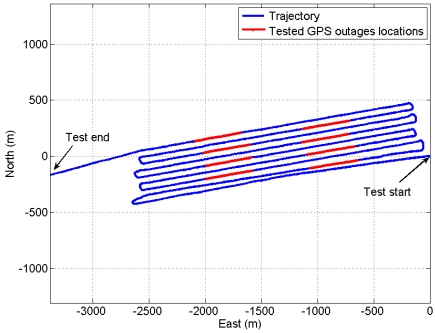
GPS test trajectory (blue) used to develop the model and GPS artificial outages (red) to test the model.

To examine the performance of the six stochastic models to be developed from the above static tests at different temperature points, dual frequency GPS data from a Trimble BD950 receiver and inertial data from the ADIS16364 IMU were collected on July 15, 2008 in Hamilton Harbour, Ontario, onboard the hydrographic surveying vessel “Merlin”, owned by the Canadian Hydrographic Service of the Department of Fisheries and Oceans. The kinematic test temperature was about 17 °C during the entire test time span. Figure 5 shows the vessel configuration. The test trajectory (blue line) with eight (8) artificial outages (each of 100 s length—red lines) is shown in Figure 6. It should be noted that two GPS antennas are used to estimate the GPS heading in addition to the GPS position solution to update the MEMS IMU navigation solution to provide accurate INS/GPS navigation solution.

## 4. Data Analysis and Results

**Figure 7.**
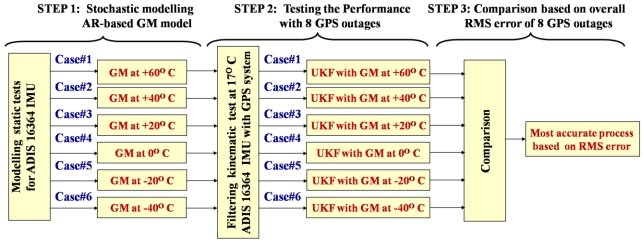
The three steps used to develop, test, and validate the AR-based GM model at different temperature points.

Figure 7 shows the three steps followed in this paper to develop one AR-based GM model per temperature point. In Step 1, static data sets are collected at different temperature points (from −40 °C to +60 °C, at 20 °C intervals) using the ADIS16364 MEMS-based IMU. In step 2, the test data collected in the kinematic mode at the specific temperature point of 17 °C are used to test the performance of the six stochastic models developed in Step 1. The integrated navigation solution from the INS/GPS is obtained using the UKF estimator. The GPS position solution from the rover GPS antenna and GPS heading solution from the two GPS antennas (vessel equipped by two GPS antennas onboard separated by 2.37 m) are employed to update the UKF filter every 1 s. UKF [also called Sigma-point KF (SPKF) in the literature such as, Wendel *et al.* [14] is used in this paper simply because the linearisation of dynamic and observation equations is not needed and the two navigation solutions of UKF and EKF are not significantly different [14]. In UKF, we use 21 inertial states (three components of each: position, velocity, attitude, gyro bias, accelerometer bias, gyro scale, and accelerometer scale errors) to develop the INS system state-space equations. Along with the state-space equations, we use the GPS positions and heading solution, and estimated INS positions and heading to develop the INS/GPS system observation equations. Then, we apply eight artificial 100 s GPS outages to test the INS-only navigation solution. In Step 3, we estimate the overall root-mean-square (RMS) error of the INS-only 3D positions and 3D orientations using the eight artificial 100 s GPS outages. Then, out of the six possible stochastic models (one for each temperature point), we select the best model, i.e., the model that exhibits the lowest RMS error, that should be applied in the UKF estimator to provide the most accurate navigation solution. It should be noted that due to the existence of high level white noise in the collected MEMS-based static and kinematic data in Steps 1 and 2, respectively, the Kaiser FIR low pass filter [8], with appropriate cut-off frequency is used to suppress this white noise. The following sub-sections show the results of the three steps shown in Figure 7 and described above.

### 4.1. Step1: AR-based GM Modelling at Different Temperature Points

The six static data sets were collected at a sampling rate of about 200 Hz at six different temperature points ranging from −40 °C to 60 °C for a period of 3 hours, which were then used to develop the six AR-based GM stochastic models (i.e., AR-based GM at −40 °C, −20 °C, 0 °C, +20 °C, +40 °C, and +60 °C) described in Section 3, for three gyro and three accelerometer bias errors. The correlation times for the AR-based GM model were estimated using Equation (10).

**Figure 8.**
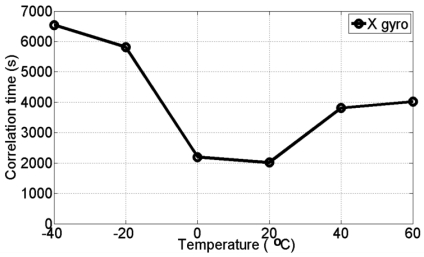

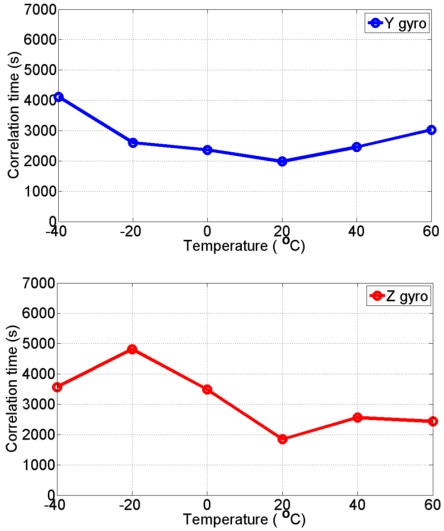
Correlation times for the three different gyros (X gyro, Y gyro, and Z gyro) at different temperature points.

**Figure 9.**
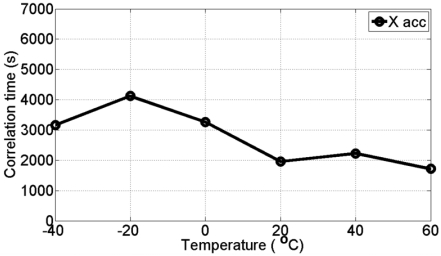

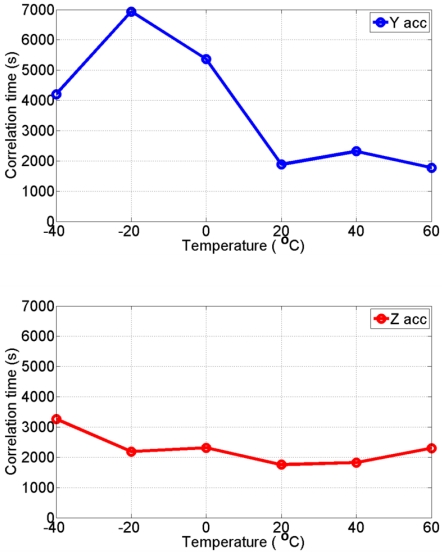
Correlation times for the three accelerometers (X acc, Y acc, and Z acc) at different temperature points.

Figures 8 and 9 show the estimated correlation times for the three gyro and the three accelerometer channels, respectively at all different temperature points. It is clear that the correlation time is temperature dependent and therefore, it is concluded that the stochastic models for MEMS-based ADIS16364 inertial sensor errors are temperature-dependent.

### 4.2. Step2: Testing the Performance of the Developed Stochastic Models

**Figure 10.**
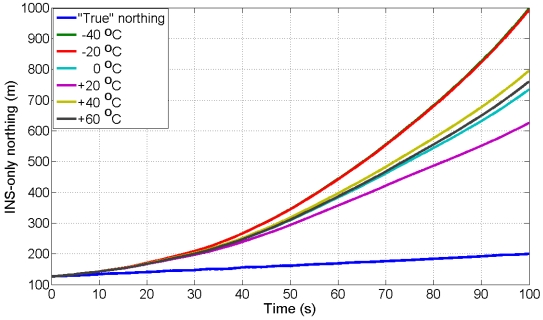
INS-only northing from kinematic test data collected at +17 °C during GPS outage#5 with AR-based GM model developed at different temperature points.

**Figure 11.**
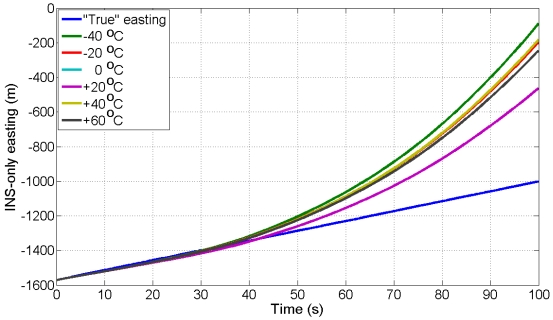
INS-only easting from kinematic test data collected at +17 °C during GPS outage#5 with AR-based GM model developed at different temperature points.

**Figure 12.**
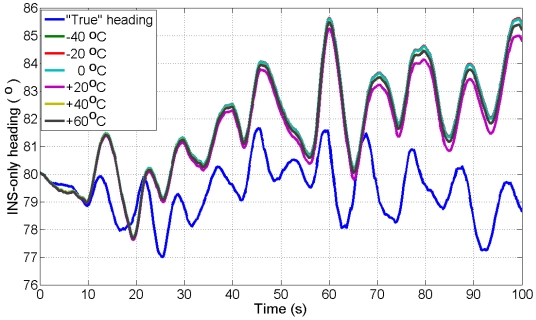
INS-only heading solution from kinematic test data collected at +17 °C during GPS outage#5 with AR-based GM model developed at different temperature points.

The UKF estimator is used to filter the kinematic data of ADIS16364 IMU mounted aboard vessel collected at +17 °C with three GPS receivers (one stationery base GPS receiver on land and two GPS receivers aboard vessel) to estimate three INS/GPS positions, three INS/GPS velocities, and three INS/GPS attitudes. The six AR-based GM stochastic model parameters (correlation times) developed in Sub-section 4.3 at different temperature points are implemented in the UKF estimator to find the stochastic model that provides the best navigation solution. To test the performance of the models, we estimate the INS-only solutions for northing, easting and heading during eight, 100 s GPS artificial outages using the UKF estimator in the prediction mode. The “true” northing and easting are estimated from two GPS receivers in differential mode (one base station GPS receiver and one rover GPS receiver aboard the vessel) whereas, the “true” heading is estimated from the two GPS antennas aboard vessel, separated by 2.37 m, as mentioned before. Figure 6 shows the locations of the eight outages (red segments). Figures 10 and 11 show an example of the performance of the INS-only solution in northing and easting, respectively during GPS outage#5. Figure 12 shows an example of the performance of the INS-only solution in heading (azimuth) during GPS outage#5. As expected, the performance in position and orientation solutions using the stochastic model developed at +20 °C (magenta) is better than the ones obtained from the stochastic models developed at the other temperature points. Similar performance is observed during the other outages and it is not shown here. The temperature dependence of the stochastic model is significant and should be considered at all times to obtain optimal navigation solution for MEMS-based INS/GPS integration.

### 4.3. Step3: Comparison Based on Overall Root-Mean-Square Error

**Figure 13.**
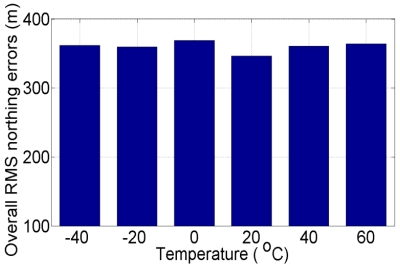
Overall RMS errors in northing using kinematic test data collected at +17 °C and the AR-based GM model developed at different temperature points.

**Figure 14.**
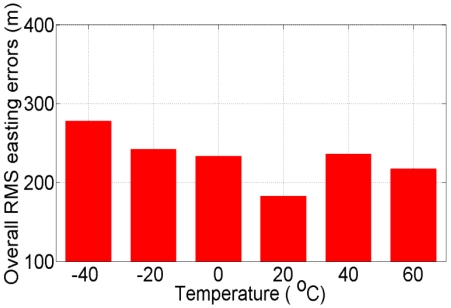
Overall RMS errors in easting using kinematic test data collected at +17 °C and the AR-based GM model developed at different temperature points.

Now, we estimate the overall root-mean-square (RMS) error for northing and easting for all eight, 100 s GPS outages. Figures 13 and 14 show the overall (average of eight GPS outages) RMS error of northing and easting respectively at different temperature points, when compared with “true” GPS-based positions.

**Figure 15.**
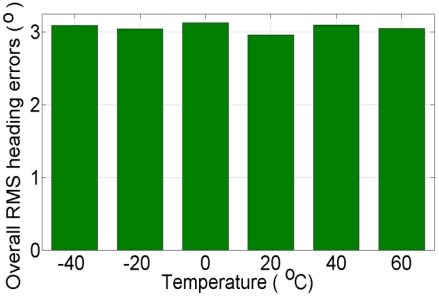
Overall RMS errors of the heading solution using kinematic test data collected at +17 °C and the AR-based GM model developed at different temperature points.

In Figures 13 and 14, the overall RMS error at +20 °C is found to be ±346.40 m and ±182.80 m for northing and easting, respectively, which is lower than the overall RMS error at all other temperature points. Figure 15 shows the overall (average of eight GPS outages) RMS error of heading at different temperature points, when compared with “true” GPS-based heading. In Figure 15 it can be seen that the overall RMS error is found to be 2.95 °C at +20 °C, which is lower than the overall RMS error of the eight GPS outages estimated with the AR-based GM model at all other temperature points.

To this end, we conclude that in order to have an optimal navigation solution, we should include in the processing stage of MEMS-based INS/GPS integration different stochastic model parameters at different temperature points with +20 °C interval and we should use the temperature-dependent stochastic model nearest to the real sensor temperature during the test.

## 5. Conclusions and Recommendations

This paper investigated the effect of changing the temperature points on the MEMS inertial sensor noise models using an AR-based GM model. The AR-based GM model estimation was achieved by using static data sets collected under different temperature points using ADIS16364 MEMS-based IMU, and showed that the estimated correlation times of an AR-based GM model for gyro and accelerometer biases are temperature-dependent. In addition, the AR-based GM models developed from stationary data sets collected at different temperature points were implemented in the UKF estimator to process and integrate inertial and GPS data collected in kinematic mode with the same inertial unit at +17 °C. The overall RMS error results from the UKF filter estimation of northing, easting and heading of eight GPS outages when compared with the “true” GPS-based position, and heading showed that the stochastic model should be developed from stationary data collected at or near the same temperature point at which the field test data were collected (i.e., stochastic model developed at +20 °C in this paper is the best model with real world kinematic data collected at 17 °C in this paper).

## Figures and Tables

**Table 1.** ADIS16364 IMU specifications [13].

***3 Gyros***	

Initial bias error	±3 °/s
In-run bias stability	0.007 °/s
Bias temperature coefficient	±0.01 °/s/ °C
Angular Random Walk	2 °/√h

***3 Accelerometers***	

Initial bias error	±8 mg
In-run bias stability	0.1 mg
Bias temperature coefficient	±0.0.05 mg/ °C
Velocity Random Walk	0.12 m/s/√h
